# Neutral evolution of drug resistant colorectal cancer cell populations is independent of their *KRAS* status

**DOI:** 10.1371/journal.pone.0175484

**Published:** 2017-10-05

**Authors:** Krastan B. Blagoev, Julia Wilkerson, Mauricio Burotto, Chul Kim, Edward Espinal-Domínguez, Pilar García-Alfonso, Meghna Alimchandani, Markku Miettinen, Montserrat Blanco-Codesido, Tito Fojo

**Affiliations:** 1 Physics of Living Systems, National Science Foundation, Arlington, Virginia, United States of America; 2 Department of Biophysics, Johns Hopkins University, Baltimore, Maryland, United States of America; 3 Medical Oncology, Center for Cancer Research, NCI, NIH, Bethesda, Maryland, United States of America; 4 Departamento de Oncologia, Clinica Alemana de Santiago, Santiago, Chile; 5 Departamento de Oncologia Medica, Gregorio Marañon University Hospital, Madrid, Spain; 6 Center for Biologics Evaluation and Research, US Food and Drug Administration (USFDA), Silver Spring, Maryland, United States of America; 7 Laboratory of Pathology, Center for Cancer Research, NCI, NIH, Bethesda, Maryland, United States of America; 8 Division of Hematology and Oncology, Department of Medicine, Columbia University, New York and James J. Peters VA Medical Center, Bronx, New York, United States of America; Seoul National University College of Pharmacy, REPUBLIC OF KOREA

## Abstract

Emergence of tumor resistance to an anti-cancer therapy directed against a putative target raises several questions including: (1) do mutations in the target/pathway confer resistance? (2) Are these mutations pre-existing? (3) What is the relative fitness of cells with/without the mutation? We addressed these questions in patients with metastatic colorectal cancer (mCRC). We conducted an exhaustive review of published data to establish a median doubling time for CRCs and stained a cohort of CRCs to document mitotic indices. We analyzed published data and our own data to calculate rates of growth (g) and regression (d, decay) of tumors in patients with CRC correlating these results with the detection of circulating MT-*KRAS* DNA. Additionally we estimated mathematically the caloric burden of such tumors using data on mitotic and apoptotic indices. We conclude outgrowth of cells harboring intrinsic or acquired MT-*KRAS* cannot explain resistance to anti-EGFR (epidermal growth factor receptor) antibodies. Rates of tumor growth with panitumumab are unaffected by presence/absence of MT-*KRAS*. While MT-*KRAS* cells may be resistant to anti-EGFR antibodies, WT-*KRAS* cells also rapidly bypass this blockade suggesting inherent resistance mechanisms are responsible and a *neutral evolution model* is most appropriate. Using the above clinical data on tumor doubling times and mitotic and apoptotic indices we estimated the caloric intake required to support tumor growth and suggest it may explain in part cancer-associated cachexia.

## Introduction

Intrinsic and acquired drug resistance, long recognized as impediments to the therapy of cancer have also emerged as problems with “targeted agents”. Described initially as acquired mutations in BCR-ABL, the target of imatinib, it was hoped it would be more straightforward than with “traditional cytotoxic agents” [1]. However, its pleiotropic nature is now well recognized and in many cases our understanding is wanting [2].

Mechanisms of resistance to “targeted therapies” include mutations in the drug target [3,4], reduced drug accumulation [5], and cellular adaptations to target engagement [6,7] among others. In the case of cetuximab and panitumumab, antibodies targeting the epidermal growth factor receptor (EGFR), prospective and retrospective analyses in patients with colorectal cancer (CRC) established the importance of *KRAS* mutations on sensitivity to these agents [8–10]. While ongoing investigations suggest some *KRAS* mutations may have less or no impact on anti-EGFR antibody sensitivity it is generally accepted patients whose tumors harbor mutant *KRAS* are less likely to benefit from anti-EGFR antibodies [11,12]. This clinical data has confirmed what was predicted nearly two decades ago–*KRAS* mutations constitutively activate the pathway through which the EGFR signals, making cells harboring activating mutations refractory to therapies that block the pathway proximal to *KRAS* [13].

While identification of mutant *KRAS* as a mechanism of *intrinsic resistance* to anti-EGFR antibodies has “personalized” this therapy and begun identifying those most likely to have better outcomes, the clinical benefit remains limited—a gain of a few months in survival—with hope better selection will bring further improvements [14–16]. The possibility an acquired *KRAS* mutation or selection of a clone harboring a *KRAS* mutation could explain acquired resistance to cetuximab and panitumumab has appeal but is theoretically unlikely. Especially in second line therapy of CRC, with median times to progression of a only a few months, it is unlikely a single cell acquiring a *KRAS* mutation or even a pre-existing clone could become a substantive portion of a tumor that even at 2 cm in diameter has approximately 8 billion cells.

Hoping to “personalize” cancer therapy and enhance our understanding of cancer biology, several groups have developed assays to quantitate circulating tumor DNA. One such effort attempted to quantitate circulating tumor DNA harboring *KRAS* mutations in patients with refractory CRC receiving panitumumab [17]. In a minority of cases, “acquired mutations” detected in serum were implicated in panitumumab resistance. This rich data set offers an opportunity to examine the likelihood *KRAS* mutations confer acquired resistance to an anti-EGFR antibody and provided a platform to examine variables affecting tumor-doubling times. We compared the growth kinetics of tumors deemed refractory to panitumumab with those of tumors in an independent cohort treated in second line. We conclude acquired *KRAS* mutations are an improbable mechanism of panitumumab tolerance. We also describe a mathematical analysis critically assessing tumor-doubling times, challenge some previous assumptions and provide insight into the caloric burden of tumors.

## Results

**Fig 1:**
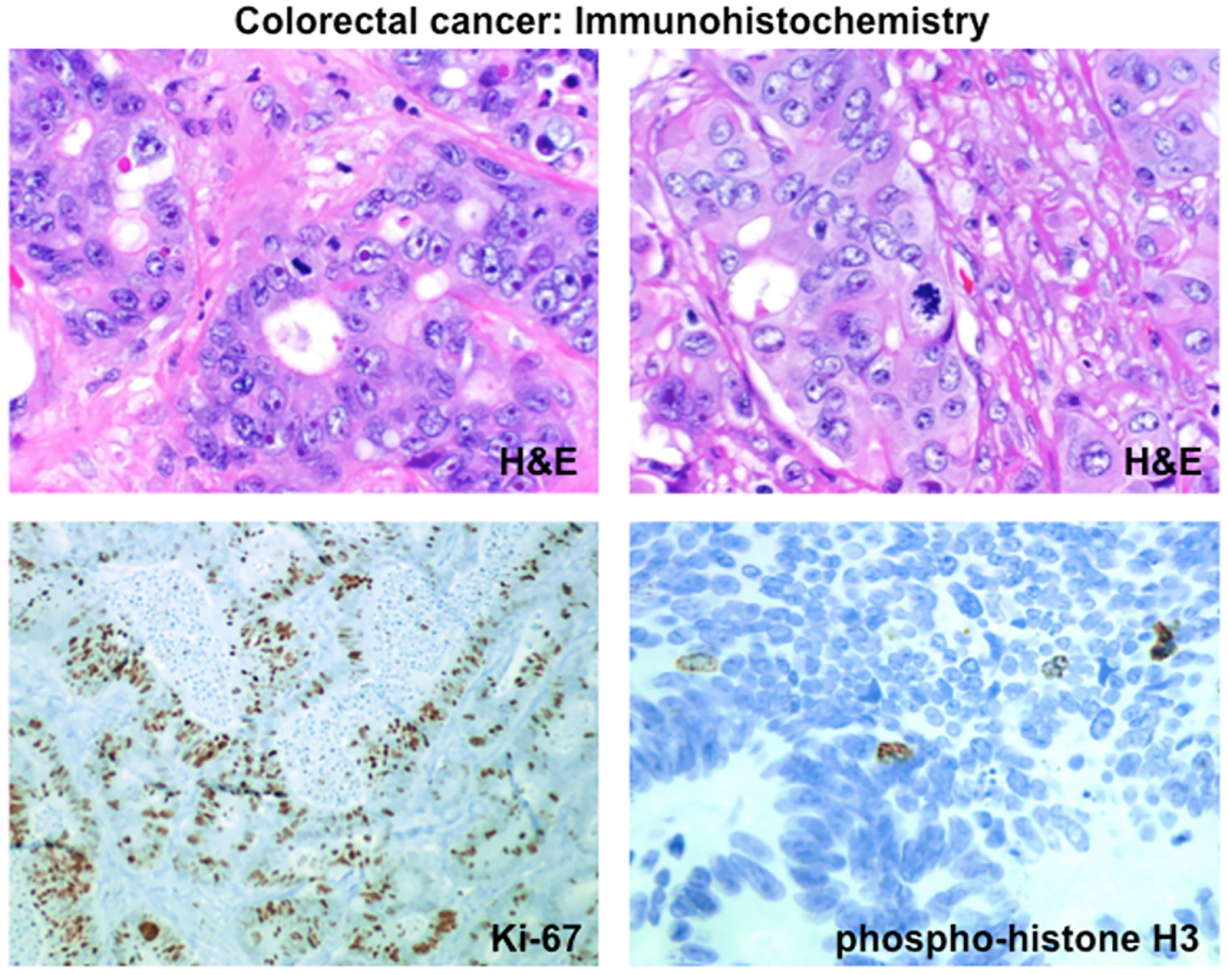
Example of a case of CRC [40X magnification]. Stains include hematoxylin and eosin (H&E), Ki-67 (MIB-1) and phosphohistone immunohistochemistry. The Ki-67 protein is associated with cell proliferation and expression can be detected during all active phases of the cell cycle (G_1_, S, G_2_, and mitosis), but is absent in resting cells (G_0_)^18^. The MIB-1 antibody was used to determine the Ki-67 labeling index. Phospho-histone H3^(Ser 10)^ is expressed in the nuclei of cells during M-phase (mitosis) allowing one to use phosphohistone staining as a means to identify cells in mitosis. H&E stain (A, x400) including atypical mitotic figures (B, x400). MIB-1/Ki-67 stain demonstrating a high proliferation index (C, x40). Phosphohistone stain demonstrating cells in mitosis [D, x400].

An exhaustive review of published data shown in Table 1, allowed us establish a median doubling time of six months for CRCs. Using radiographic measurements, the doubling times of CRCs in patients range from 2.9 to 31.0 months (median 6.8 months). Consistent with this, we found experimentally in thirteen CRCs as shown in Fig 1 and Table 2, a median of only eight mitosis/HPF and ***0*.*3% of all cells in mitosis***. This was further corroborated by phosphohistone staining, a marker of mitosis that stained very few cells in these CRCs. Importantly in these thirteen samples 40–70% of cells stained positive for MIB-1/Ki-67. The monoclonal antibody, MIB-1, was developed using recombinant portions of the Ki-67 nuclear antigen as immunogen. As such MIB-1 recognizes Ki-67, which is associated with cell proliferation and is found throughout the cell cycle (G1, S, G2, and M phases) but not in resting (G0) cells. The MIB-1 / Ki-67 proliferation index is defined as the percent of immunoreactive tumor cells in the evaluated area. The high MIB-1 indices relative to the very low percent of malignant cells in mitosis as summarized in Table 2 underscores the fact MIB-1/Ki-67 staining, is a “marker of proliferation” and not a surrogate for cell division [18].

**Table 1:** Doubling times of colorectal carcinomas.

Author (year)	Clinical Presentation	Time (months)
**Choi et al (2013)**	Ascending colon	21.6
	Transverse colon	8.4
	Descending colon	6
	Sigmoid	18
	Rectum	9.6
**Kim et al (2012)**	Pulmonary metastasis	5.3
**Sadahiro (2005)**	Pulmonary metastasis	2.1
**Umetani et al (2000)**	Non pedunculated colorectal carcinomas	6.8
**Matsui et al (2000)**	Colorectal carcinoma mucosa	31
	Colorectal carcinoma submucosa	25
	Colorectal carcinoma muscularis propria	13
	Colorectal carcinoma serosa	13
**Finlay et al (1988)**	Overt liver metastasis	5.1
	Occult liver metastasis	2.9
**Tada et al. (1984)**	Colon Carcinomas	3 to 34
	Adenomatous polypus	4.8 to 13.2
**Bolin et al. (1983)**	Colon and rectal carcinoma	4.3
**Range**		2.9–31
**Median** (using lowest values for two reports with a range)		6.8

**Table 2:** Tabulation of mitosis in colorectal cancers.

CRC Sample	Mitoses per 10 HPF	Percent of malignant cells in mitosis	Ki-67 / MIB-1 proliferation index
1	8	0.3%	50 − 60%
2	23	0.7%	60%
3	4	0.3%	50 − 60%
4	3	0.1%	30 − 40%
5	6	0.3%	60 − 70%
6	20	0.9%	60 − 70%
7	5	0.2%	50 − 60%
8	6	0.2%	40 − 50%
9	30	0.7%	70%
10	9	0.3%	60 − 70%
11	9	0.3%	50 − 60%
12	5	0.2%	40 − 50%
13	12	0.3%	60 − 70%
**Median**	**8**	**0.3%**	**55%**
**Range**	**3–30**	**0.1%—0.9%**	**30–70%**

**Fig 2:**
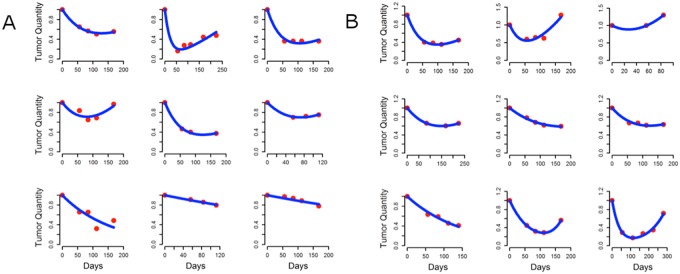
Evolution of tumors in patients is shown as graphs of RECIST measurements. **A**: Nine patients with detectable MT *KRAS* DNA in serum. **B**: Nine patients without detectable MT *KRAS* DNA in serum. As can be seen the kinetics of tumor regression and growth of these tumors is indistinguishable. Detection of a mutant *KRAS* DNA is serum, interpreted as evidence of “acquiring” this genetic alteration, does not impact the growth of the tumor. The blue lines are the lines drawn by the model and this demonstrates how well the theoretical (blue line) fit the actual (red symbols). The fit of the actual data to the theoretical had p values less than 0.05 in all cases, and in most much less than that.

**Fig 3:**
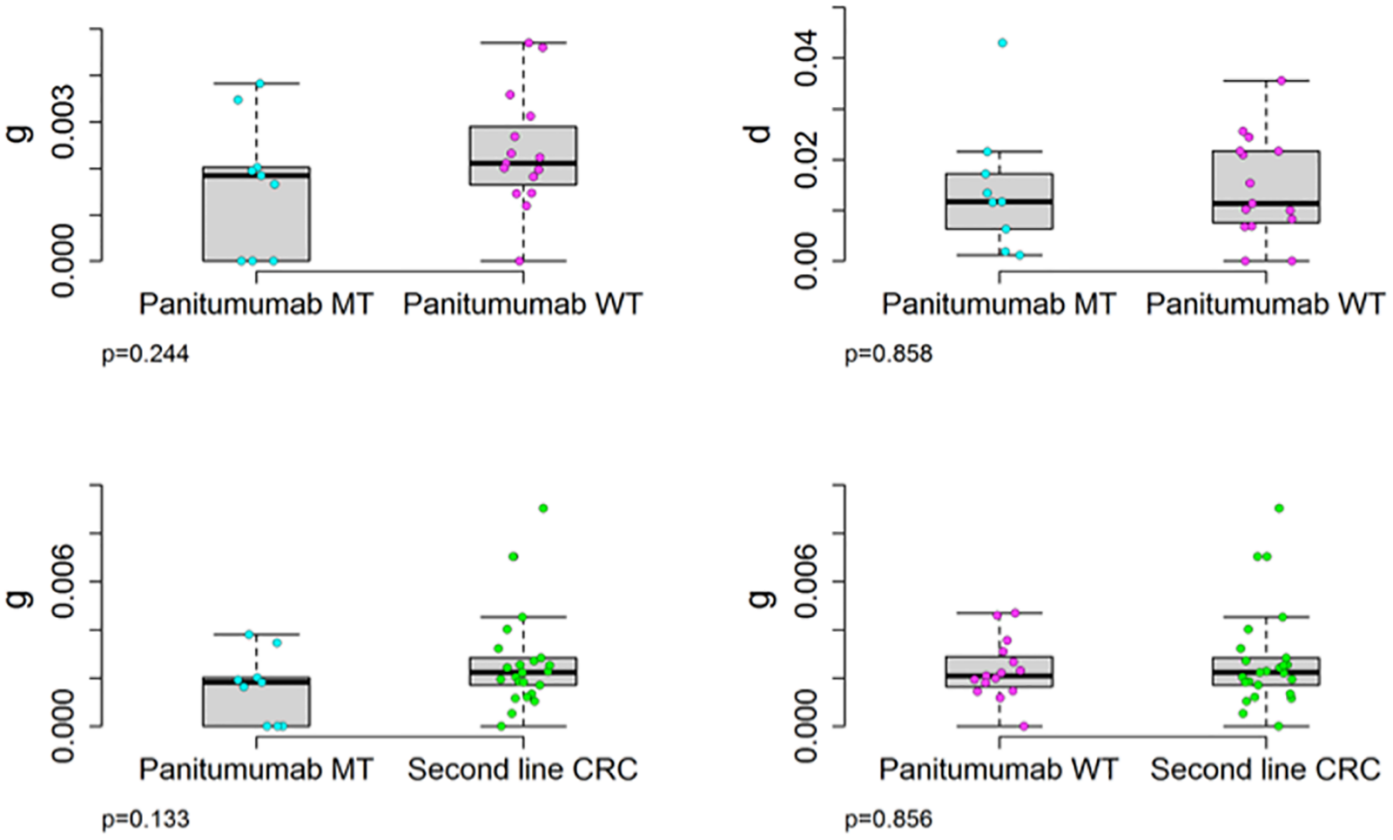
Comparison of growth rate (*g*) using tumor measurements from Diaz et al [17] and from an independent CRC tumor data set of patients treated in second line with conventional chemotherapy. **Top 2 panels:** The growth (g, p = 0.244) and regression (*d*, p = 0.858) rates of tumors treated with panitumumab in which mutant *KRAS* DNA could be detected in serum (MT) do not differ from those of tumors without detectable mutant *KRAS* DNA in serum (WT). **Lower 2 panels:** Compared to tumors independently treated in second line with conventional chemotherapy the growth rates (*g*) of the panitumumab-treated tumors is similar for both the subgroup with detectable circulating mutant *KRAS* DNA (MT) in serum and those without detectable mutant *KRAS* DNA in serum (WT).

Next we analyzed published data on 24 patients with CRC treated in second line with panitumumab-containing regimens [17] comparing this data with that in an additional 22 patients with CRC treated in second line with similar oxaliplatin or irinotecan-containing regimens with/without anti-EGFR antibodies. The published and our own clinical data allowed us to calculate rates of growth (*g*) and regression (*d*, decay) of tumors in these patients with a methodology validated using measurements of tumor quantity from >6000 patients with diverse cancers treated with various chemotherapeutics [19–22]. These analyses have demonstrated the quantity of tumor measured is the sum of the tumor quantity that is regressing and is sensitive to therapy plus the tumor quantity that is relatively resistant and growing. Published data on 24 patients with CRC whose tumors were initially *KRAS*-WT, reported circulating MT-*KRAS* DNA as “newly detected” in nine treated in second line with panitumumab plus chemotherapy [15]. Using the actual published data of serial tumor quantities [17] we calculated the growth (*g*) and regression (*d*) rates for the individual tumors. We did this by fitting the data, as shown in Fig 2, and readily calculating the growth rate (g) of tumors in the nine patients with newly detected circulating MT-*KRAS* DNA (Fig 2A). As shown in Fig 3, we determined the growth rate of tumors in the nine patients with newly detected circulating MT-*KRAS* DNA to be 0.0019 days^-1^. This value was statistically indistinguishable [p = .244] from the growth rate of 0.0021 days^-1^ calculated for tumors in the fifteen patients without detectable circulating MT-*KRAS* DNA (Fig 2B). Importantly, we further compared the growth rates of these tumors to those of tumors in patients receiving conventional second line therapies both with/without anti-EGFR antibodies. The average growth rate of these twenty-two tumors, 0.002 days^-1^, was statistically indistinguishable from the growth rate calculated for the tumors treated with panitumumab—p values of .306 and .769 for comparisons with those with/without circulating MT-*KRAS* DNA, respectively. Furthermore using published serial CEA values in a subset of the patients treated with panitumumab [17], similar results were obtained with estimated growth rates of 0.0031 days^-1^ and 0.0021 days^-1^ in tumors with/without circulating MT-*KRAS* DNA, respectively [p = .1265]. We also found statistically indistinguishable (p = .858) rates of tumor regression for tumors with/without circulating MT-*KRAS* DNA, 0.0117 days^-1^ and 0.0114 days^-1^, respectively (Fig 3). Finally, using a mathematical analysis (S1 File) we show that the *KRAS* mutant and *KRAS* wild type populations evolve together with little evidence of competition and with the same growth rates.

Although the foregoing established that selection of a mutant *KRAS* clone cannot explain acquired resistance to an anti-EGFR antibody the analysis brought into focus how such a clone could emerge, specifically how fast a tumor has to divide and what can be sustained. Because these calculations are relevant to other situations where clonal overgrowth might be considered, we sought to determine mathematically using our own and published data what would occur and the caloric requirements needed to sustain tumor growth. In this regard, if one knows the mitotic index, the average times for mitosis and apoptosis, and the actual tumor doubling times measured clinically, an apoptotic rate can be inferred allowing one to estimate the tumor quantity lost due to cell death as tumors grow (S1 File, S1–S3 Data)

Tumor doubling times measured clinically using radiologic imaging or serum markers—a value we will term the *clinical tumor doubling time* (CTDT)–are much longer than one would estimate using the mitotic index obtained histologically (Table 2). The large number of cells that die concurrently explains the difference. Using the measured mitotic index we can calculate first what would be the tumor doubling time in the absence of apoptosis, a value we will call the minimal doubling time (MDT). We distinguish the MDT from the actual CTDT that as will be seen is always a larger value because while some tumor cells divide, others die. Using our measured mitotic index of 0.3%, and a 2-hour value for the duration of mitosis or the time during which cells are counted as “mitotic” and contribute to the mitotic index [23,24], we obtain 19.8 days as the MDT (S1 File). This is to be contrasted with a value of four days used by others [17,25], a value more appropriate for mouse colonic crypts. For the MDT to be four days (in the absence of apoptosis) the mitotic index has to be higher (1.6%) than the one we observed (0.3%). But as we have noted, apoptosis occurs concurrently with mitosis, and one can calculate the apoptotic index that together with a given mitotic index will achieve actual CTDTs (S1 File, S1–S3 Data). Estimates of the time spent in apoptosis [26–28] allow one to estimate an apoptotic index, the percent of cells in apoptosis at any time. Thus, for example, with a CTDT of 60 days, a mitotic index of 0.3%, 2-hours spent in mitosis, and 2-hours as the time spent in apoptosis we obtain an apoptotic index of 0.19%. By comparison if one assumes a MDT of four days [17,23] the apoptotic index must be 1.2% (S1 File, S1–S3 Data).

**Fig 4:**
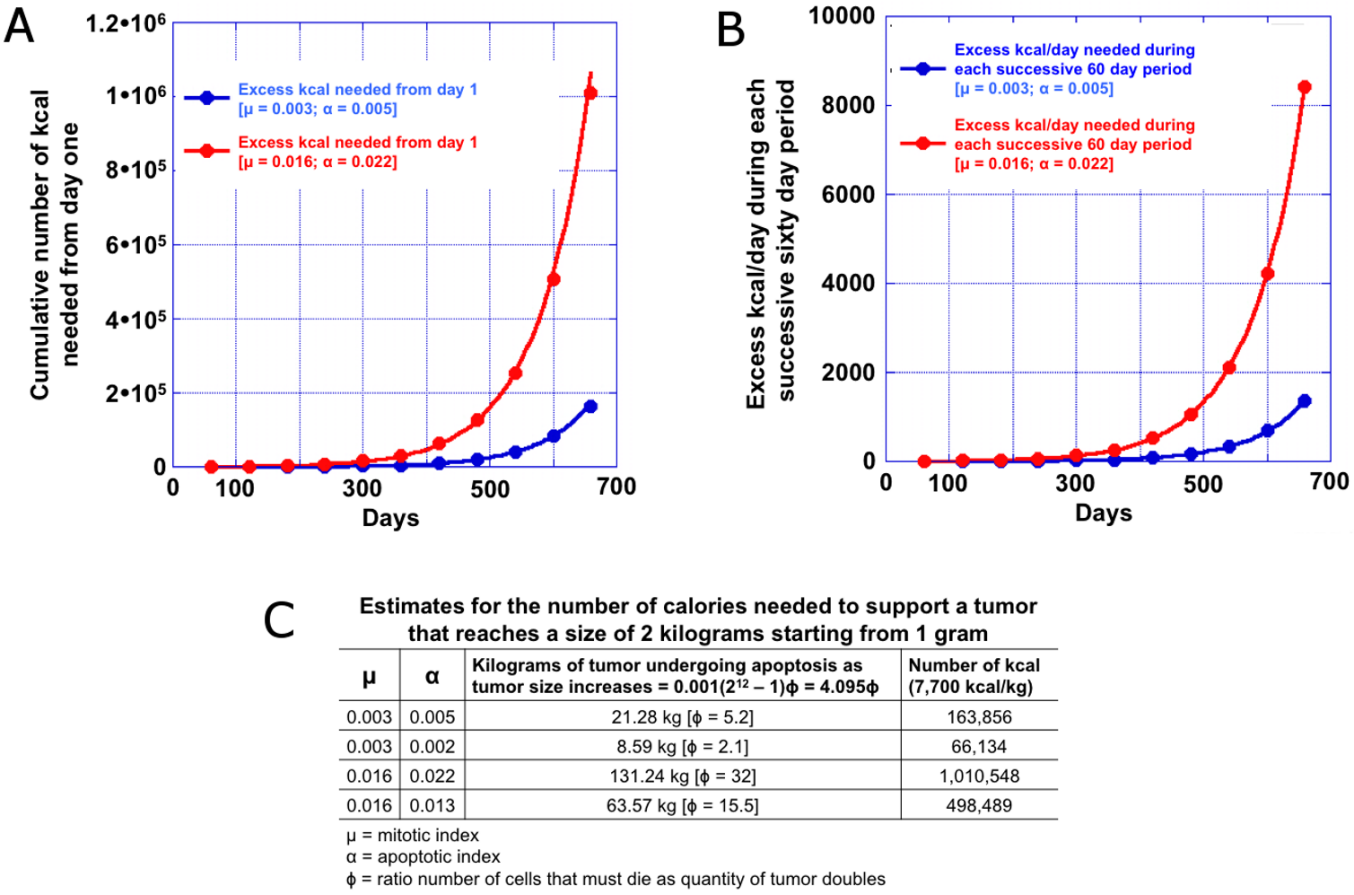
Estimates (**A**) the cumulative number of additional kilocalories and (**B**) the number of additional kilocalories per day required by a tumor for each successive 60-day doubling period required due to the tumor. The estimates are for two different mitotic and apoptotic indexes. The doubling time is 60 days and the tumor mass progresses through 11 doublings. The estimates include the number of calories to produce both the cell that survive, as well as those that undergoes apoptosis. (**C**) Estimates for the number of calories needed to support a tumor.

**Fig 5:**
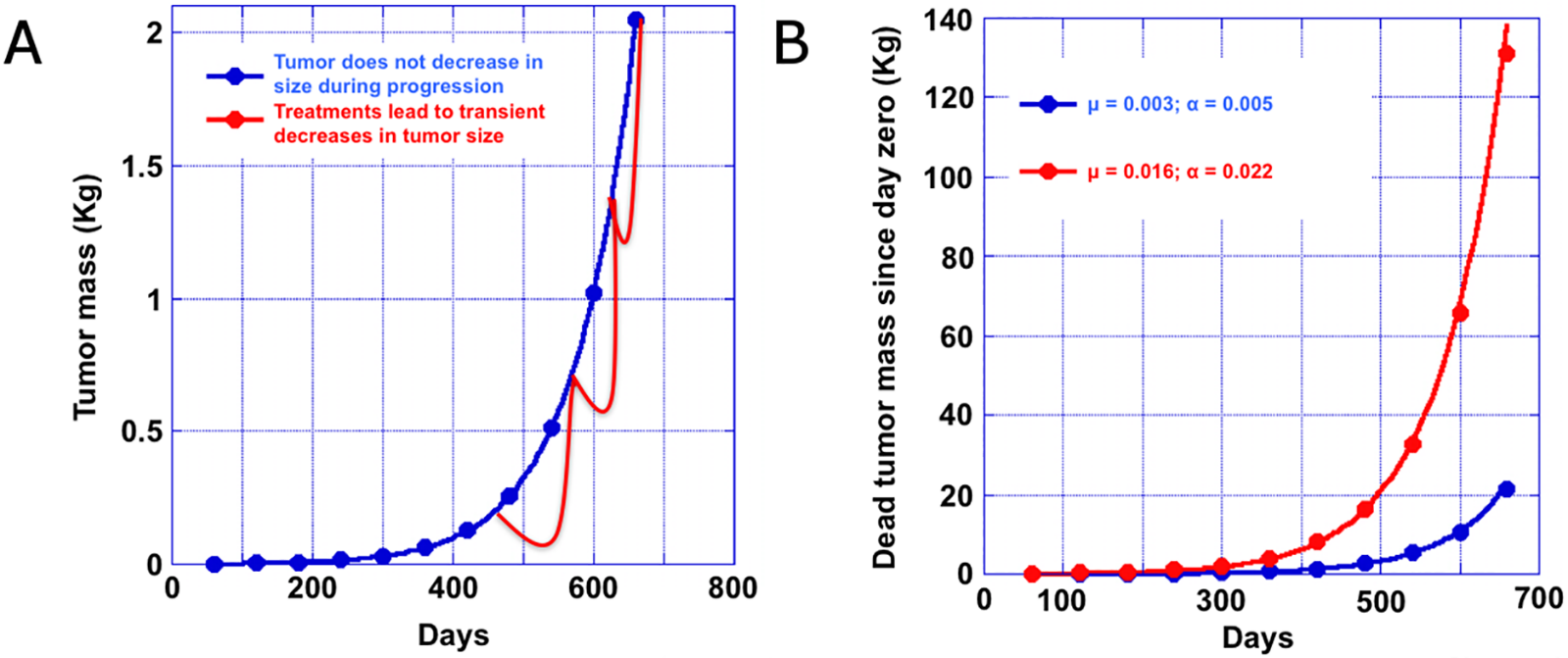
(**A**) Progression of tumor quantity from 1 gram to 2 kilograms with a doubling time of 60 days. The blue curve depicts a tumor that does not decrease at all during progression, while the red curve depicts an example where intermittent treatments lead to transient decreases in tumor size. Resistance then emerges and the tumor again beings to grow. Thus the blue model that we use provides estimates for the minimum amount of new tumor formed and the minimum caloric requirements. (**B**) Total amount of tumor that undergoes apoptosis as the net tumor quantity increases from 1 gram to 2 kilograms for two different mitotic (μ) and apoptotic (α) indexes. The doubling time is 60-days and the tumor mass progresses through 11 doublings.

Given the calculations above that establish the apoptotic index one can estimate the ratio of cells that die to new cells arising from mitosis. We will use the term ϕ for the ratio of the number of cells that must die as the quantity of tumor doubles (the CTDT). Note that in this analysis the number of cell divisions needed to double the quantity of tumor is independent of tumor size. Using our mitotic index of 0.3% we determine that ϕ is ≈2. By comparison if one assumes the MDT to be 4 days, ϕ increases to 15. In the first example with ϕ≈2, as a tumor doubles from 0.5 kg to 1kg, one kg of tumor is lost (dies) as 0.5 kg of new “viable” tumor is added. With ϕ≈15 as the tumor doubles from 0.5 kg to 1 kg, 7.5 kilograms of tumor are lost (die) as 0.5 kg of new “viable” tumor is added. That is to say, 8 kg of tumor are produced and only 0.5 kg survive, while the other 7.5 kg die. Given that it takes ≈7,700 kcal to create a kg of tissue with a ϕ≈2 an additional ≈11,550 kcal [7,700 x 1.5] calories will need to be consumed during the CTDT– 60-days for CRC—to produce the viable and dead tumor, an average of ≈200 extra kcal/day. Most of these calories would be required towards the end of the 60-day CTDT, because the number of dying cells scales as the power of number of days (Supporting Information). In the case of a MDT of 4 days the number of additional calories needed would be ≈61,600 or ≈1,027 kcal per day. In Fig 4, we depict the additional calories a patient must consume to support tumor growth with estimates provided in Fig 4C for the number of calories needed in the case of different mitotic indices, assuming at the time of death the quantity of tumor has reached 2 kg, a quantity that would take 11 doublings to reach starting from 1 gram of tumor. In Supporting Information we show that the total number of dead cells is Δ = N_0_ϕ(2^J+1^–1), where the constant factor ϕ relates the initial number of cells and the number of cells that die during doubling of the tumor. The number of cells that die after 11 doublings is Δ = 4095N_0_ϕ. Starting with one gram of tumor, which has approximately a billion cells, we see that the total mass of tumor that dies as the cancer grows is Δ = 4.095ϕ kg. The factor ϕ is a very important parameter reflecting the tumor biology and prognosis in every patient. In Supporting Information we calculate ϕ for different mitotic rates. In Fig 5A, we plot the viable tumor mass depicting a tumor that does not decrease at all during progression, and one where intermittent treatments lead to transient decreases in tumor size. In the latter as resistance emerges the tumor grows. Note that the calculations in Fig 4 do not involve any decrease during progression and thus provides estimates for the minimum amount of new tumor formed and the minimum caloric requirements. Fig 5B depicts the quantity of tumor that undergoes apoptosis during this period based on the two different estimates of the MDT (our estimate of 19.8 and the value of 4 days claimed by others).

While these estimates capture the growth phase of tumors, most patients are under treatment during disease progression and for at least part of the treatment period the tumor mass decreases in time as depicted in Fig 5A. We show this to underscore that our estimates do not include the receding phases of the tumors and thus underestimate the calories needed during actual treatment.

## Discussion

We embarked on this analysis to better understand resistance to a targeted therapy. Although *acquired mutations* have been identified in patients receiving BCR-ABL and BRAF inhibitors, such mutations have not been described with VEGF or mTOR inhibitors amongst others, suggesting mechanisms other than mutations in the target or its pathway might be equally if not more important. Given the detailed understanding of the EGFR pathway and clinical data demonstrating a lack of efficacy for EGFR blockade in cells harboring *KRAS* mutations, published data offered a valuable model to explore the importance of mechanisms of resistance [17]. From our analysis we conclude that as regards *KRAS* in metastatic colorectal cancer (mCRC) treated with an anti-EGFR antibody a *neutral evolution model* and not *KRAS* mutations provides a greater understanding of the clinical outcome.

The *neutral theory of molecular evolution* posits that most genetic variation results from random mutations and genetic drift and not from natural selection. Because such *neutral mutations* do not affect the ability to survive or reproduce, the fitness of any given cell in a population is unaffected by the version of the gene a cell harbors. Neither the published data nor our data is sufficient to conclude acquired resistance to the anti-EGFR antibody panitumumab is due to the emergence of cells harboring mutant *KRAS*. While circulating MT-*KRAS* was detected in the serum of some patients, given known tumor heterogeneity, it would not be surprising that the occurrence and expression of mutant *KRAS* in some tumors is heterogeneous. This would lead to detection of circulating mutant *KRAS* as tumor burden increased and all cells increased in number including those harboring wild-type or mutant-*KRAS*. In this case an assay detecting circulating mutant *KRAS* simply detects more tumor; it does not explain drug tolerance.

Furthermore, in patients with MT-*KRAS* in the serum, the growth rate remained constant even as circulating MT-*KRAS* was initially detected or appeared to increase, suggesting (1) the detection or increase in mutant *KRAS* templates was inconsequential, and (2) not only the growth rate of the entire tumor was constant but cells harboring MT-*KRAS* had no impact on the tumor’s growth rate nor were likely growing faster. As regards the latter, one would expect cells that might be resistant to grow or accumulate faster and in turn accelerate the rate of tumor growth. Indeed with a stated conversion factor of 44 million *KRAS*-mutant cells for every mutant *KRAS* template/ml [17], the estimated MT-*KRAS* tumor burden ranged from 0.13 grams to 21 grams, with 7/9 patients with circulating MT-*KRAS* having <5 grams of tumor harboring a *KRAS* mutation. These values, easily estimated using published data [17], represent only a very small fraction of the tumor burden and could not meaningfully contribute to tumor growth in these patients with refractory CRCs who likely have several hundred grams or more of tumor. Such small quantities may also explain the fluctuations in circulating mutant *KRAS* seen with the data. Thus, one can confidently conclude mutant *KRAS* did not confer a growth advantage and leads us to conclude that as regards agents targeting the EGFR a neutral evolution model best explains the data.

A possible reason for the neutral evolution of different cell populations is that by the time a patient presents with advanced disease, the various cell populations in a cancer have undergone a prolonged selection and have similar growth rates. As mutations are only important if they confer an advantage—in this case the ability to grow better during panitumumab therapy—one can further conclude detection of such mutations was inconsequential. However, the data is inconclusive whether MT-*KRAS* cells are/are not resistant to panitumumab and whether they co-exist with other resistant clones.

As we conducted these analyses we also realized very little attention has been given to the simultaneous occurrence of mitosis and apoptosis in tumors. Previous studies that have reported “b ≈0.25 for colorectal cancer cells”, corresponding to one cell division every 4 days” [17,23] are difficult to reconcile with the literature summarized in Table 1 and with our own estimate of tumor-doubling times of ≈130 days using linear RECIST dimensions, and assuming the tumors approximated spheres. In this case a 27% increase in the linear dimension (1.27 compared to original) would double tumor volume with an estimated tumor-doubling time of ≈130 days:

Doublingtime=[ln1.27/Tumorgrowthrateconstant]=[0.239/0.0019]=126days

A similar value was estimated using CEA as the measure of tumor quantity or volume. Additionally we would note that one cell division every four days means a patient presenting with a single 2 cm tumor—a minimal amount for a patient with metastatic CRC—would have more than 2 kilograms of tumor one month later and more than 500 kilograms at two months—unless more than 99% of the cells underwent apoptosis, a highly unlikely proposition confirmed by examining any mCRC histologically and finding, as shown in Fig 1, primarily viable not necrotic or apoptotic cells therein.

Finally, we realized the data provided us the opportunity to address the extent to which apoptosis occurs and provide some estimates of caloric consumption needed to support tumor growth. Cancer cachexia (CC) is characterized by energy imbalance, due to increased energy expenditure and/or reduced energy intake reduced [29]. Although poor appetite with diminished energy intake plays an important role in CC, this alone cannot account for weight loss in cancer patients [30], a thesis supported by the fact that intensive nutritional support such as total parenteral nutrition fails to reverse CC [31]. This suggests increased energy expenditure and metabolic derangements are likely important features of CC. A number of studies have demonstrated resting energy expenditure (REE) is increased in various cancers including lung, and gastrointestinal cancers [32–35]. Patients with stage IV cancer have higher REE compared to those with lower stage disease [35], while patients with non-small cell lung cancer with elevated REE who undergo curative resection have a decrease in REE [36]. Finally in patients with mCRC similar to those in this study, other investigators using computed tomography imaging data and a mathematical model concluded increases in REE may contribute substantially to CC-associated weight loss. Specifically they noted that increases *in the mass and proportion* of high metabolic rate tissues, including liver and tumor, led to a cumulative incremental REE of 17,700 kcal during the last 3 months of life, estimates similar to ours and not consistent with 4-day doubling times [37]. We would note that in principle it might be possible to address this issue in a trial, however it might be impractical given that the patient’s weight is a complex function of their metabolism, type of treatment administered and the tumor’s response to the treatment, supportive measures and other factors.

The low mitotic indices we observed indicate only a minority of cells are “in cycle” at any one time. But with doubling times of 60 days clinically—values in the low range of reported—a substantial amount of cell death occurs and indeed more tumor dies than is newly generated suggesting *tumor growth is relatively inefficient*. Using our most conservative estimates it becomes apparent that as cancers grow an increasing number of calories are required to support tumor growth, amounts that can exceed several hundred calories per day. In a cancer patient with a reduced appetite this could contribute to the problem of CC. As to whether some of the caloric requirements be satisfied by autophagy, we think this unlikely [38–40]. As our understanding of this lysosomal degradation process evolves, it is increasingly clear its principal functions are not to provide calories but rather to adapt to stress, maintain cellular homeostasis, and promote survival. Furthermore, it would not be energy efficient or an energy-free source of calories, and would likely only satisfy a small fraction of the patient's needs.

In summary, we conclude *KRAS* mutations are insignificant as a mechanism of resistance to EGFR blockade. A neutral evolution model and not *KRAS* mutations provides a greater understanding of the clinical outcome. Whether novel strategies designed to enhance the efficacy of EGFR blockade will be effective remains to be determined [41]. We also find that despite low mitotic indices and relatively “long” tumor doubling times tumors impose a substantial caloric burden on patients.

## Materials and methods

### Data

The data used had either been previously published and available in the public domain or was obtained from patients enrolled in studies or receiving standard of care therapy at Gregorio Marañon University Hospital. The studies on which patients were enrolled had the approval of the Gregorio Marañon University Hospital IRB and the Agencia Española del Medicamento and informed written consent was obtained from all patients. As we analyzed data regarding the effect of various drugs but did not change a patient’s treatment (retrospective / observational study) the analysis was exempt by the Agencia Española del Medicamento.

### Mathematical analysis

Our regression-growth equation sees the change in tumor quantity during therapy resulting from 2 independent component processes (both following first order kinetics): an exponential decay/regression, *d*, and an exponential growth/regrowth of the tumor, *g*. Eq 1 is:

f(t)=exp(-d•t)+exp(g•t)-1 (1)

Where the tumor quantity (f, sum of LDs) for a given time t (days), is a non-linear function of *d* (decay), *g* (growth) and time (t). All tumor quantities are divided by 1, the assigned tumor quantity at time zero; exp is the base of the natural logarithms. For data showing a continuous decrease from start of treatment, *g* is eliminated:

f(t)=exp(−d•t) (2)

Similarly, when tumor measurements show a continuous increase, *d* is eliminated:

f(t)=exp(g•t) (3)

### Data analysis

We modeled each data set. Parameters were estimated with associated Student *t* and *P* values and predicted values were obtained using SAS software and the procedure NLIN, a non-linear regression model.

### Immunohistochemistry

Cases of metastatic colorectal cancer were selected and pathology reviewed on paraffin-embedded, hematoxylin and eosin stained sections, with special attention to mitotic activity and proliferation index. Immunohistochemistry for Ki-67 (Dako, Product no. M7240, dilution 1:200) was performed using a Ventana Benchmark autostainer (Ventana Medical Systems, Tucson, AZ). Phosphohistone immunostains were performed using Phospho-Histone H3 (Ser28) Antibody from Cell Signaling (Danvers, MA). Mitotic index (MI) represents the number of mitotic figures per 10 high power fields (hpf); the number of mitotic figures was counted in a larger area of the tumor (total of 10 hpf) and does not take into account the tumor cell density. The percent mitoses were calculated as the number of mitotic figures per 100 cells (estimated using an ocular grid). As in many cancers, the MIB-1/Ki-67 proliferation index varied in different areas of the tumor. Tumor areas with the highest density of positively staining nuclei were identified on low power scanning and these areas were used for estimating percent positive nuclei for the MIB-1/Ki-67 proliferation index.

## Supporting information

S1 File (DOCX)

S1 Data (XLSX)

S2 Data (XLSX)

S3 Data (XLSX)
