# Supplementary material for: Neutral evolution of drug resistant colorectal cancer cell populations is independent of their KRAS status
Source: PLoS One. 2017 Oct 5;12(10):e0175484. doi: 10.1371/journal.pone.0175484 (PMC5628783; doi:10.1371/journal.pone.0175484)
Supplement: S1 File — (DOCX) [file pone.0175484.s001.docx]

**SUPPLEMENTAL INFORMATION**

A. Growth of Drug Resistant and Drug Sensitive Cell Populations

While a patient is treated with panitumumab, the number of tumor cells at any instant of time is represented by:

$$N_{p}=N_{pR}+N_{pS}$$

Here, N_pR_ represents the number of cells that are resistant to panitumumab and N_pS_ that are sensitive to panitumumab. Similarly during chemotherapy the number of tumor cells are represented by:

$$N_{c}=N_{cR}+N_{cS}$$

In previous work we have shown that in many different cancers tumor kinetics can be described by simple mathematical model, which for a patient being treated with panitumumab is:

$$N_{p}(t)=N_{pR}^{0}e^{gt}+N_{pS}^{0}e^{-dt}$$

and for a patient on chemotherapy is:

$$N_{c}(t)=N_{cR}^{0}e^{Gt}+N_{cS}^{0}e^{-Dt}$$

In this formula $N_{pR}^{0}$ and $N_{pS}^{0}$are the number of resistant and sensitive cells respectively at the beginning of the panitumumab therapy and similarly $N_{cR}^{0}$ and $N_{cS}^{0}$are the number of resistant and sensitive cells respectively at the beginning of chemotherapy. In most patients during treatment the tumor quantity initially decreases, reaches a minimum after which it enters a growth phase. During the growth phase the terms $N_{p/cS}^{0}e^{-dt}$ describing the sensitive cells is negligible compared to the growth term of the resistant cells and we will ignore them in our further analysis. In the growing phase thus the tumor growth is described by:

$$N_{p}(t)=N_{pR}^{0}e^{gt}$$

and

$$N_{c}(t)=N_{cR}^{0}e^{Gt}$$

In a cohort of patient with CRC there are two types of patients. In some of the patients there is no detectable population with MT-KRAS cells and in the others a detectable MT-KRAS cells are present. This means that the growth of tumor for the patients treated with panitumumab can be represented as:

$$N_{p}\left( t \right)=N_{pR}^{0MT}e^{g_{MT}t}+N_{pR}^{0WT}e^{g_{WT}t}$$

where $N_{pR}^{0MT}$ and $N_{pR}^{0WT}$ are the number of MT-KRAS and WT-KRAS cells at the beginning of treatment. We do not expect WT-KRAS cells to have different behavior from MT-KRAS cells during chemotherapy. Our experimental data showing similar growth rate of tumors during panitumumab and chemotherapy lead to the conclusion that:

$$N_{p}\left( t \right)=N_{pR}^{0MT}e^{g_{MT}t}+N_{pR}^{0WT}e^{g_{WT}t}={\alpha N}_{cR}^{0}e^{Gt}{=\alpha N}_{c}(t)$$

where α is a proportionality constant. However, if the only cells resistant to panitumumab harbor MT-KRAS then:

$$N_{p}\left( t \right)=N_{pR}^{0MT}e^{g_{MT}t}={\alpha N}_{cR}^{0}e^{Gt}{=\alpha N}_{c}(t)$$

and since the tumors during these two different treatments grow with the same growth rate i.e. g_MT_ = G. The only conclusion that can be drawn from this is that the cells initially resistant to both treatments are proportional to each other during the entire course of treatment with a constant of proportionality.

B. Cell Division and Cell Death

Here we answer the question: Given a measured mitotic index (μ) = 0.3% = 0.003, time in mitosis (m) = 2 hours, how these parameters relate to the doubling time of the tumor and the apoptotic index (α). In a tumor, not all cells are “in cycle”. However, once a cell enters the cell cycle it will traverse through the cell cycle. The number of different “sets” of cells that go through mitosis within a 24-hour period is 24 divided by 2 or 12 different sets of cells. This can be understood if one thinks of what one is looking at in a stained slide. When one stains a section of a tumor and finds that 0.3% of all tumor cells are in mitosis, all one knows is that the mitotic index is low (in this case 0.3%). But since mitosis lasts at most 2 hours, had the tumor been removed two hours later these cells in which mitotic figures were observed would have finished their mitosis and would not have been “caught in mitosis”. Instead another 0.3% of cells would have been found to be in mitosis. Thus every two hours a different 0.3% of the cells would be in mitosis. At the end of a 24-hour day 3.6% of the cells would have gone through mitosis. If at any one time the fraction of cells that are in mitosis is 0.003 (0.3%), the fraction of cells that will divide in a 24-hour period will be 12 X 0.003 = 0.036 (3.6%). So that in this 24-hour period 3.6% of the cells will be dividing. Were no cell death to occur, the number of cells at the end of 24 hours would be 103.6% of the original. After an additional 24 hours the number would then be 103.6 X 1.036 = 107.3% of the original number and so on. Indeed as we will see below, if cell death did not occur, after about 20 days the number would be about 200% and the tumor would have doubled in size. But as some cells in a tumor are dividing we know also that a fraction of the cells are undergoing spontaneous apoptosis and are dying − with the fraction/percent of cells undergoing this spontaneous apoptosis to be referred to as the apoptotic index. If the initial tumor is composed of N_0_ cells we will calculate, on average what the apoptotic index must be if the tumor doubles in M number of days, at which time the tumor will then have 2N_0_ cells.

First the general relationships between these parameters are derived within the framework of a simple mathematical model and then specific examples are analyzed.

The main goal is to evaluate the number of cells that will die in the time it takes the tumor to double in quantity. The mitotic time will be denoted by **m** [hours], the mitotic index by **μ** [percent or fraction], the apoptotic index by **α** [percent or fraction], and the apoptotic time by **a** [hours]. In a tumor not all cells are “in cycle”; some cells are in G_0_. The fraction of cells in the tumor that pass through mitosis during a day is pN with p = μ24/m and the fraction of cells that go through apoptosis is (α24/a) N = qN, where N is the cells that are present. **Therefore the parameters that control the quantity of tumor are μ/m and α/a.** The precise evaluation of these ratios is very important for predicting tumor evolution. Our experience with tumor growth is that the tumor kinetics during disease progression in humans obeys first order kinetics and follows simple exponential laws, i.e. the classic Skipper laws (Skipper HE: Perspectives in cancer chemotherapy: Therapeutic design. Cancer Res 1964; 24:1295-1302; Skipper HE: Laboratory models: Some historical perspective. Cancer Treat Rep 1986; 70:3-7.). This suggests that μ/m and α/a do not change during disease progression and thus can be taken as constant. After a day has elapsed the new number of cells is:

N_1_ = (N_0_ - pN_0_) + 2pN_0_ - q[(N_0_ - pN_0_) + 2qN_0_]

= N_0_ + pN_0_ - q(N_0_ + pN_0_) = N_0_(1 + p)(1 − q)

After the second day the number of cells is:

N_2_ = N_1_(1 + p)(1 − q) = N_0_[(1 + p)(1 − q)]^2^

After n days have passed the number of cells will be:

N_n_ = N_0_[(1 + p)(1 − q)]^n^

The condition that determines the number of days required for doubling of a tumor is:

N_n_ = N_0_[(1 + p)(1 − q)]^n^ = 2N_0_

or

[(1 + p)(1 − q)]^n^ = 2

which leads to the relation:

n = ln2 / [ln(1 + p) + ln(1 - q)]

Here the property of the logarithmic function ln[(1 + p)(1 - q)] = ln(1 + p) + ln(1 - q) was used. Next the notation y = 1 + p and z = 1 - q will be used.

Next we compute how many cells die during each tumor doubling. The number that dies during the first tumor doubling (D_1_) is:

D_1_ = q(N_0_ + pN_0_) = N_0_q(1 + p)

As shown above:

N_1_ = N_0_(1 + p)(1 − q)

and after the second day the number of cells that have died (D_2_) is:

D_2_ = q(N_1_ + pN_1_) = N_1_q(1 + p) = N_0_q(1 - q)(1 + p)^2^

N_2_ = N_0_(1 + p)^^(1 − q)^2^

and after the third day the number of cells that have died (D_3_) is:

D_3_ = q(N_2_ + pN_2_) = N_2_q(1 + p) = N_0_q(1 - q)^^(1 + p)^3^

After n days have passed the number of cells that have died (D_n_) is:

D_n_ = q(N_n-1_ + pN_n-1_) = N_n-1_q(1 + p) = N_0_q(1 - q)^n-1^(1 + p)^n^

Therefore the number of cells that die in n days after which the tumor is doubled is:

Δ_1_ = D_1_ + D_2_ + D_3_ + … + D_n_

= qN_0_(1 + p) + qN_0_(1 - q)(1 + p)^2^ + qN_0_(1 – q)^2^(1 + p)^3^ + … + qN_0_(1 - q)^n-1^(1 + p)^n^

= qN_0_(1 + p){1 + (1 - q)(1 + p) + (1 - q)^2^(1 + p)^2^ + … + (1 - q)^n-1^(1 + p)^n-1^}

Using the formula for the sum of a geometric series:

1 + r^2^ + r^3^ + … +r^n-1^ = (1 – r^n^) / (1 – r)

We conclude that the total number of cells that die during the first tumor doubling time is

Δ_1_ = qN_0_ + p)[1 – (1 - q)^n^( + p)^n^] / [1 – (1 – q) + p]

= qN_0_y(1 – (yz)^n^) / (1 – yz).

This result means that during each tumor doubling when the number of cells goes from N to 2N cells the number of cells that die is [qy(1 – (yz)^n^)/(1 – yz)]N = φN, where the factor φ = [qy(1 – (yz)^n^)/(1 – yz)]. The factor φ does not depend on the number of cells and thus it is a universal number during tumor progression. This of course is oversimplification since during tumor progression and treatment the apoptotic index changes and so does the mitotic index. However to illustrate our point we will assume that they do not. If during disease progression the tumor goes through J number of doublings the total amount of tumor that will die will be

Δ = Δ_1_ + Δ_2_ + Δ_3_ + … + Δ_J_

= N_0_φ + N_1_φ + N_2_φ + … + N_J_φ

= N_0_φ + 2N_0_φ + 4N_0_φ + … + 2^J^N_0_φ

= N_0_φ(1 – 2^J+1^) / (1 – 2)

Thus the total number of tumor cells that will be lost during tumor progression consisting of J tumor doublings is:

Δ = qN_0_y(1 – (yz)^n^) / (1 – yz)(2^J+1^ – 1) = N_0_(2^J+1^ – 1)φ.

Here p = μ24/m, q = (α24/a), y = 1 + p, and z = 1 – q.

The way to use this formula is to first calculate n, the number of days necessary for tumor doubling from the formula:

n = ln2 / ln(1 + μ24/m)(1 - α24/a)

and then calculate Δ by using n and the experimental measurements of α, a, μ, m, and J. Another use of this formula is from the mitotic index μ and the observed doubling time τ to calculate the apoptotic index α from the formula:

ln(1 + p)(1 - q) = (ln2)/n

1 - q = exp(ln2/n) / (1 + p)

α = (a/24)(1 - exp(ln2/n) / (1 + p))

Again from α, a, μ, m, and J one can estimate Δ.

Now we discuss an example. With the numbers quoted at the beginning of this supplement the percent of cells that will divide during the first cell cycle is N_0_x12x0.003 = 0.036N_0_. First we will assume that the apoptotic index is 0. Thus after the first day there will be N_1_ cells:

N_1_ = (N_0_ - 0.036N_0_) + 2N_0_ = N_0_ + 0.036N_0_ = N_0_(1 + 

Here, we had to subtract the number of dividing cells from the initial number, because the rest of the cells are not dividing and only these cells will double during the first day. The second day we start with N_1_ cells and we end with:

N_2_ = (N_1_ - 0.036N_1_) + 2N_1_ = N_1_(1 +  = N_0_(1 + ^2^

After m divisions the number of cells will be:

N_n_ = N_0_(1 + ^n^

Now we can ask how many days n are needed to double the initial tumor. The condition for m is:

N_n_ = N_0_(1 + ^n^ = 2N_0_

After dividing by N_0_ we obtain:

(1 + ^n^ = 2

Taking the ln on both sides leads to:

n ln(1 +  = ln2

n = ln2 / ln((1 +  = 0.693 / 0.035 = 19.8 days

The above then is the number of days after which the tumor quantity has doubled assuming that there has been no cell death occurring concurrently.

However, as our review of the literature has shown, the observed clinical tumor doubling time (CTDT) is not 20 days, but rather it is 60 days. So next we ask how many cells will have to die to shift the tumor doubling time from 20 days to 60 days?

We will assume that the time a cell spends in apoptosis is 2h. One can be critical of our use of an apoptotic index, but for our rough estimates it is a usable concept. Actually our estimates of the apoptotic index and the duration of apoptosis are arbitrary as the literature on this is imprecise due to the fact that these estimates are very dependent on the context and the assays utilized. However, this is inconsequential since what we are estimating is actually the death index. The latter is the number of cells that must die per day, given a known mitotic index, such that the net tumor growth coincides with the measured CTDT. Because the duration of time during which apoptosis is detectable morphologically will vary depending on the method of assessment used this influences the apoptotic index. If the apoptotic change that one is detecting lasts a long time, the apoptotic index will be higher, “even though the actual number of cells undergoing apoptosis would be the same. Because of this, the apoptotic index cannot be correlated with an actual death index” [Reference 25 in the text]. However, the product: [apoptotic index] x [24 hours divided by the duration of apoptosis] is constant – designated (α24/a) above. Thus assuming the time a cell spends in apoptosis is 2h, the different set of cells that will go through apoptosis is 12 in 24 hours. We will denote by α the apoptotic index. After the first day we will have N_1_ cells i.e.:

N_1_ = (N_0_ - 0.036N_0_) + 2N_0_ – 12α((N_0_ – 0.036N_0_) + 2N_0_)

= N_0_ + 0.036N_0_ – 12α(N_0_ + 0.036N_0_) = N_0_(1 + (1 – 12α)

We subtract the percent of cells that will go through apoptosis in one day i.e. 12α(the number of new cells) = 12α(N_0_ + 0.036N_0_). On the second days we obtain:

N_2_ = (N_1_ - 0.036N_1_) + 2N_1_ – 12α((N_1_ – 0.036N_1_) + 2N_1_)

= N_1_(1 + (1 – 12α) = N_0_(1 + ^2^(1 – 12α)^2^

After m divisions the number of cells will be:

N_m_ = N_0_[(1 + (1 – 12α)]^m^

Now we can ask what the apoptotic index has to be for the tumor to double in 60 days. The condition for α is:

N_m_ = N_0_[(1 + (1 – 12α)]^60^ = 2N_0_

Canceling N_0_ on both sides and taking the ln we obtain:

60ln[(1 + (1 – 12α)] = ln2

ln[(1 + (1 – 12α)] = ln2/60 = 0.012

(1 + (1 – 12α) = exp(0.012) = 1.012

(1 – 12α) = 0.977

α = 0.0019 = 0.19%

How many cells will die in total in 60 days? After the first cell division the number of dead cells is:

D_1_ = 12α(N_0_ + 0.036N_0_)

After the second day the number of dead cells is:

D_2_ = 12α(N_1_ + 0.036N_1_) = 12αN_1_(1 + 0.036) = 12αN_0_(1 + ^2^(1 – 12α)

After the third day the number of dead cells is:

D_3_ = 12α(N_2_ + 0.036N_2_) = 12αN_2_(1 + 0.036)

= 12α(1 + 0.036)(N_0_(1 + ^2^(1 – 12α)^2^)

= N_0_12α(1 + ^3^(1 – 12α)^2^

Thus at the end of the 60^th^ day the number of cells that died on that day will be:

D_60_ = 12α(N_59_ + 0.036N_59_) = N_0_12α(1 + ^60^(1 – 12α)^59^

The total number of cells that died in 60 days is:

D = D_1_ + D_2_ + D_3_ + … + D_60_ =

= N_0_12α(1 + ^1^(1 – 12α)^0^ + N_0_12α(1 + ^2^(1 – 12α)^1^ + … + N_0_12α(1 + ^60^(1 – 12α)^59^

= N_0_12α(1 + [1 + (1 + ^1^(1 – 12α)^1^ + (1 + ^59^(1 – 12α)^59^]

= N_0_12α(1 + [(1- (1 + ^60^(1 – 12α)^60^) / (1- (1 + (1 – 12α))]

We used the formula for the sum of a geometric series:

1 + r^2^ + r^3^ + … +r^n-1^ = (1 – r^n^) / (1 – r)

Using the numbers from our example we obtain

D = N_0_ 0.0228x1.036 [(1 – 8.348x0.251) / (1 – 1.036x0.9772)]

= N_0_0.0236x1.0954/0.0124 = 2.032N_0_

This means that in 60 divisions 2g of tumor will die in order to produce an additional 1g of viable tumor.

Now we can assume that the doubling time is 4 days without apoptosis. How much tumor will die in 60 days, the observed CTDT? For the doubling time to be 4 days in the absence of apoptosis the mitotic index has to be higher than the one we observed. To see what it has to be we use the formula from above:

(1 + 12μ^4^ = 2

μ = 0.016 = 1.6%

Thus 12μ = 0.189 and the apoptotic index has to satisfy:

(1 + 189(1 – 12α) = 1.012

α = 0.012 = 1.2%

So we have 1 + 12μ = 1.189, 1 – 12α = 0.856 and the total number of dead cells is:

D = N_0_0.144x1.189[(1- 1.189^60^0.856^60^) / (1- 1.189x0.856)]

= N_0_0.17 (1 - 2.8796) / (1 - 1.018) = N_0_17.75

This shows that if the doubling time without apoptosis is 4 days in order for the tumor to double in 60 days (the know CTDT) 18g of tumor must die or 18 times the initial amount of tumor. If the initial tumor is 1 kg then 18kg of tumor will be made and have to die for the tumor to add 1kg and double in 60 days.

| **μ** | **m**  **[h]** | **α** | **a**  **[h]** | **Doubling time [days]** | **Disease progression time [days]** | **φ = [(α24/a)y(1 – (yz)^n^) / (1 – yz)]** |
| --- | --- | --- | --- | --- | --- | --- |
| 0.003 | 1 | 0.005 | 2 | 60 | 660 | 5.20 |
| 0.003 | 2 | 0.002 | 2 | 60 | 660 | 2.10 |
| 0.016 | 1 | 0.022 | 2 | 60 | 660 | 32.05 |
| 0.016 | 2 | 0.013 | 2 | 60 | 660 | 15.52 |

| Initial tumor quantity = 0.001kg (1 gram; ≈1 cm diameter tumor) | | |
| --- | --- | --- |
| **Number of Tumor Doublings** | **φ = [(α24/a)y(1 – (yz)^n^)/(1 – yz)]** | **Kilograms of tumor undergoing apoptosis as tumor size increases**  **0.001(2^12^ – 1)φ = φ** |
| 11 | 5.20 | 21.28 kg |
| 11 | 2.10 | 8.59 kg |
| 11 | 32.05 | 131.24 kg |
| 11 | 15.52 | 63.57 kg |

| This is **Figure 5C** from the main text | | | |
| --- | --- | --- | --- |
| **μ** | **α** | **Kilograms of tumor undergoing apoptosis as tumor size increases = 0.001(2^12^ – 1)φ = φ** | **Number of kcal (7,700 kcal/kg)** |
| 0.003 | 0.005 | 21.28 kg | 163,856 |
| 0.003 | 0.002 | 8.59 kg | 66,134 |
| 0.016 | 0.022 | 131.24 kg | 1,010,548 |
| 0.016 | 0.013 | 63.57 kg | 504,889 |
| μ =   α =   | | | |
